# Opportunistically collected data from aerial surveys reveal spatio-temporal distribution patterns of marine debris in German waters

**DOI:** 10.1007/s11356-020-10610-9

**Published:** 2020-09-07

**Authors:** Bianca Unger, Helena Herr, Sacha Viquerat, Anita Gilles, Patricia Burkhardt-Holm, Ursula Siebert

**Affiliations:** 1 Institute for Terrestrial and Aquatic Wildlife Research, University of Veterinary Medicine Hannover, Foundation, Werftstraße 6, 25761 Büsum, Germany; 2 Center of Natural History (CeNak), University of Hamburg, Martin-Luther-King-Platz 3, 20146 Hamburg, Germany; 3 Department of Environmental Science, Man-Society-Environment, University of Basel, Vesalgasse 1, 4051 Basel, Switzerland

**Keywords:** Aerial surveys, Marine pollution, Nature conservation, Monitoring, Offshore and coastal waters

## Abstract

Marine debris is known for its ubiquitousness and harmful effects on marine life. This study is the first analysis to provide information on the distribution of floating marine debris in German waters using aerial survey data collected between 2002 and 2016. During regular harbour porpoise monitoring flights, 191,167 km were covered and 26,512 floating debris items recorded (average encounter rate 0.1387 items/km). Debris was encountered more often in the North Sea than in the Baltic Sea (0.16 items/km; 0.08 items/km). The average encounter rate was higher in offshore waters than in coastal areas. Overlaps of marine debris distribution with ‘Special Areas of Conservation’ are a particular reason for concern. Moreover, the spring months (March–May) were identified to be the time of the year with the highest average encounter rates for marine debris. Fishing-related debris was shown to contribute up to 25% of the total number of all observed items. This study shows that opportunistically collected data on marine debris from aerial surveys are valuable for identifying distribution patterns of floating debris without additional survey effort and costs. These data can be used as baseline information to inform management schemes such as the Marine Strategy Framework Directive.

## Introduction

Marine debris is a growing problem in all the world’s oceans (Barnes et al. 2009; Galgani 2015; Pham et al. 2014).

**Fig. 1:**
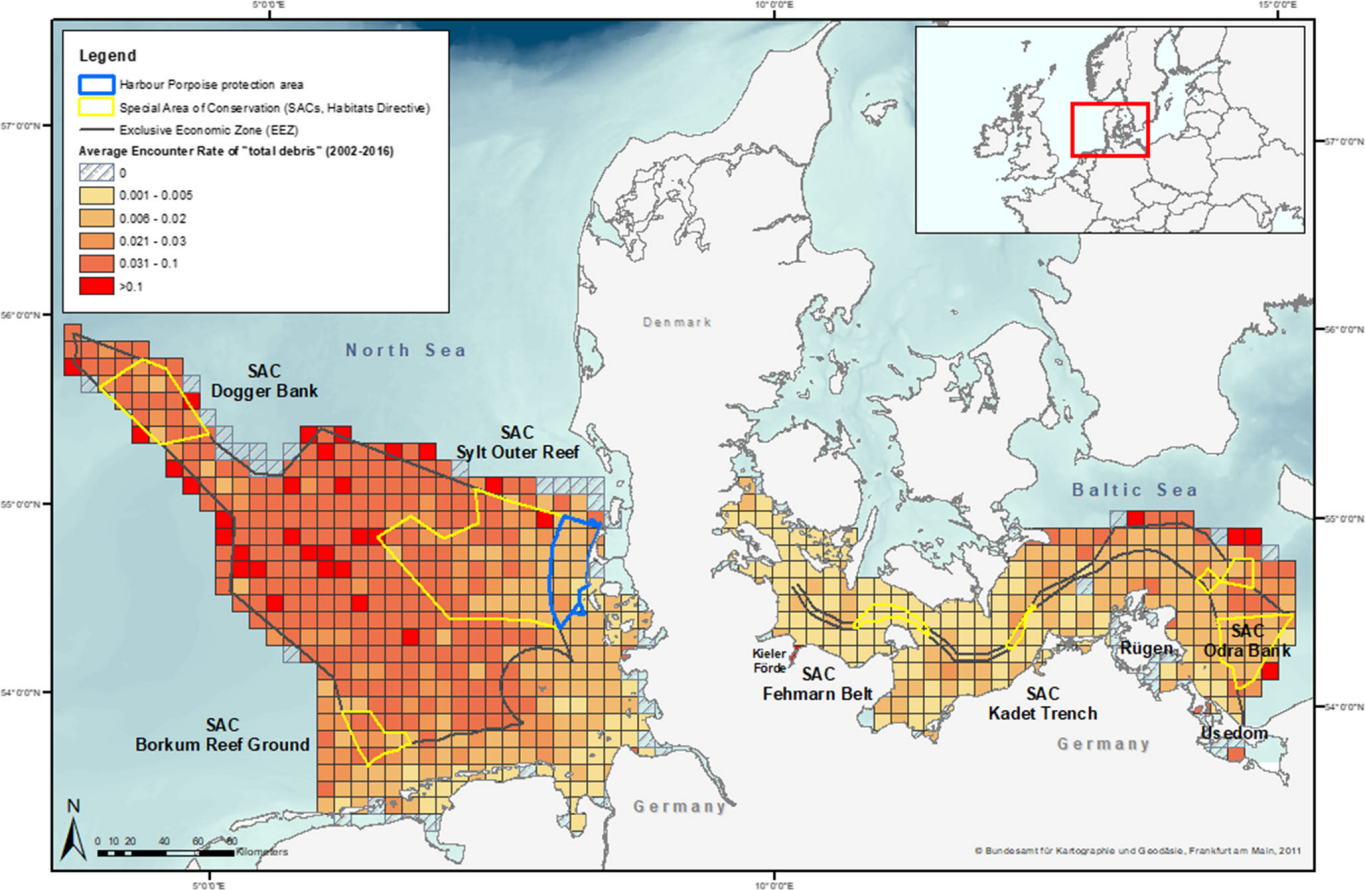
Marine debris distribution in the German North Sea and Baltic Sea presented as average encounter rate (AER, total debris) per grid cell (10 × 10 km) (aggregated data from 2002 to 2016, grid provided by the European Environmental Agency, https://www.eea.europa.eu). Basemap provided by Bundesamt für Kartographie und Geodäsie, Frankfurt am Main 2011

**Fig. 2:**
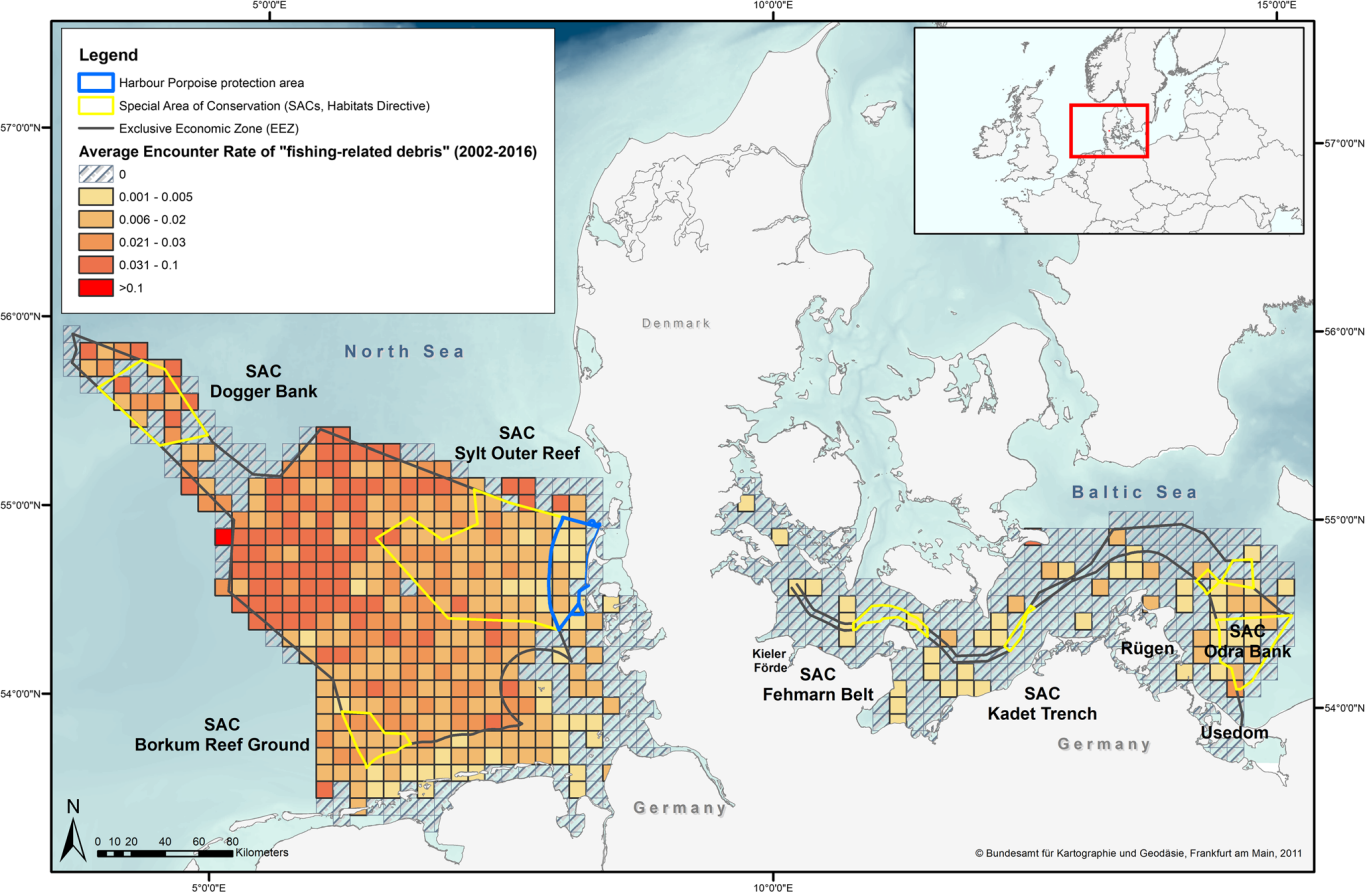
Fishing-related debris distribution in the German North Sea and Baltic Sea presented as average encounter rate (AER, fishing-related debris) per grid cell (10 × 10 km) (aggregated data from 2002 to 2016, grid provided by the European Environmental Agency, https://www.eea. Basemap provided by Bundesamt für Kartographie und Geodäsie, Frankfurt am Main 2011

While a large proportion of marine debris sinks to the sea floor (70% of all marine debris), 15% of marine debris is found on beaches, and 15% is floating on the surface (UNEP 2005). Winds and currents transport floating marine debris over large distances, causing local aggregations as well as dispersal (van Sebille et al. 2012). Due to their low density and high durability, especially plastic items are the major contributor to floating marine debris (Hammer et al. 2012; Katsanevakis 2008). It is estimated that approximately 5 trillion plastic items with a total weight of > 250,000 t are currently floating in the world’s oceans (Eriksen et al. 2014). Besides deteriorating the quality of marine habitats (Moore 2008; Vegter et al. 2014), the occurrence of marine debris is associated with a variety of impacts on marine wildlife, including fish, sea turtles, sea birds, pinnipeds and cetaceans (Camphuysen 2008; Foekema et al. 2013; Kühn et al. 2015; Laist 1987). Floating marine debris poses a high risk of entanglement and ingestion for marine predators feeding in the water column or at the surface (Allen et al. 2012; Baulch and Perry 2014; Derraik 2002; Gregory 2009; Jacobsen et al. 2010; Jepsen and de Bruyn 2019; Kühn et al. 2015; Simmonds 2011). Thus, particular attention must be paid to the areas that have been established for nature conservation (Figs. 1 and 2). The Special Areas of Conservation (SACs) in German waters belong to the Natura 2000 network under the auspice of the Habitats Directive (Habitats Directive 1992) and require a special assessment. The same is true for the harbour porpoise protection area in the North Sea (Figs. 1 and 2).

Information on floating marine debris is particularly important in the context of marine conservation and management. In Europe, marine debris is considered to be harmful according to the Marine Strategy Framework Directive (MSFD, 2008/56/EC) and constitutes one of the descriptors for establishing a ‘good environmental status’ (GES): The ‘descriptor 10’ requests that ‘properties and quantities of marine litter do not cause harm to the coastal and marine environment’ (Marine Strategy Framework Directive 2008).

For assessing the environmental status, information on debris loads and its spatial distribution are required. A number of ship-based surveys have been conducted in European waters to assess floating marine debris distribution (Aliani et al. 2003; Sá et al. 2016), also in the North Sea and Baltic Sea (Dixon and Dixon 1983; Gutow et al. 2018; Rothäusler et al. 2019; Thiel et al. 2011). However, the spatial extents of these surveys were limited, providing information only for selected areas. Large-scale information on marine debris distribution in European waters is lacking. So far, aerial surveys have not been considered as a source of information on floating marine debris, because dedicated aerial surveys are comparatively expensive and detection of objects is limited to items larger than 30 cm (Galgani et al. 2013). Using aerial photography for estimating abundances and densities of marine debris is questionable at this stage, since the method lacks validation compared with observer-based surveys (Garcia-Garin et al. 2019).

At the same time, large-scale surveys for marine mammals and sea birds are regularly conducted in European waters. As implied by Galgani et al. (2013), using established aerial surveys as a monitoring method for floating debris is a valuable way of observing marine debris distribution, especially because they enable data collection at no additional cost or time effort. However, information on debris is rarely recorded during these surveys and seldom analysed.

In Germany, a monitoring scheme for assessing the distribution and abundance of harbour porpoises (*Phocoena phocoena*) has been in place since 2002 (Gilles et al. 2011; Gilles et al. 2009). Aerial surveys have been conducted regularly since then, covering the entire German North Sea and Baltic Sea, collecting sighting data on marine mammals. As part of the survey protocols, sightings of marine debris have been recorded in a standardised manner during all surveys. These data have not yet been evaluated. However, the collection of data on marine debris has not been the focus of these aerial surveys and must be considered a by-product with associated limitations, i.e. lack of specific strip width estimation and prioritisation of collection of marine mammal data. Still, these data represent a potentially valuable source of information on floating marine debris distribution, evaluated for the first time in the course of this study. Thereby, this study is the first to use this extensive dataset to explore the occurrence of surface-floating macro debris in German waters.

Here, we analyse this dataset to explore the applicability and limitations of opportunistically collected data on marine debris from aerial surveys. With this evaluation, we aim to investigate what information can be derived from a ‘side product’ of marine top predator surveys and to gain a first overview of marine debris distribution in German waters by (i) roughly estimating the amount of floating marine debris, as well as conducting both (ii) a spatial and (iii) a temporal analysis.

## Material and methods

### Study area

This study evaluated data from aerial surveys covering the German North Sea (NS) and German Baltic Sea (BS). The North Sea, a marginal sea of the north-east Atlantic Ocean, covers an area of 750,000 km^2^ and is connected with the Baltic Sea in the east through the Skagerrak (OSPAR 2000). The Baltic Sea is a semi-enclosed sea with a size of 420,000 km^2^ (HELCOM 2018). German marine waters (i.e. the 12-nm Zone and the Exclusive Economic Zone, EEZ) cover an area of 56,541 km^2^ (NS 41,034 km^2^; BS 15,507 km^2^) both in coastal and offshore waters (BfN 2018). Forty-five percent of German waters are currently defined as areas in need of special protection (BfN 2018) (Figs. 1 and 2).

### Aerial surveys

Aerial surveys dedicated to observing marine mammals have regularly been conducted in the area since 2002 in order to estimate the abundance and distribution of harbour porpoises (*Phocoena phocoena*). These surveys followed the standard ‘line transect distance sampling’ methodology (Buckland et al. 2001), flown at a constant altitude of 600 ft. and with ground-speeds of 90–100 kts (165–185 km/h). Two bubble windows on the right and left sides of the airplane enable the two observers to monitor the water surface directly under the aircraft. The observation is carried out with the naked eye. All relevant information on sightings is reported to a navigator (data collector) via an intercom system. The navigator enters the information into a computer so that all data are directly digitally available. The computer is connected to a portable GPS device. The location is saved automatically by the system every 2 s. This enables to record the position of all sightings precisely. In addition to the sightings, information on environmental conditions (e.g. sea state, turbidity) is noted. Further details on the protocol of marine mammal monitoring using aircrafts are provided in Scheidat et al. (2008) and were revised in Gilles et al. (2009) and Hammond et al. (2013). Transects were designed perpendicular to the assumed gradient of harbour porpoise density distribution according to distance sampling methodology (Buckland et al. 2001). During these surveys, sightings of floating marine debris were also recorded. These records included time and position of the marine debris sighting, and a binary categorisation into (i) general debris (household debris, industrial debris, plastics, processed wood etc.) and (ii) fishing-related debris (nets, ropes, fishing gear, buoys etc.). Opportunistically, detailed specifications of detected items such as material, size or specific identification (e.g. plastic box, blue plastic foil) were documented. Items from the size of a regular milk carton (TetraPak ©, 23 cm × 7 cm) upwards were recorded.

The total survey effort was 191,179 km (German NS: 137,161 km; German BS: 54,018 km) in the period between 2002 and 2016 (Table 1).

**Table 1:** Effort in km per year and season

Year	Season				*Total*
	Spring	Summer	Autumn	Winter	
German North Sea (survey effort in km)					
2002	940	5019	2366	0	8325
2003	3354	3223	2282	629	9486
2004	341	3663	3596	284	7884
2005	5382	3938	4510	881	14,710
2006	5773	2793	259	0	8825
2007	1008	1685	0	0	2693
2008	2081	2754	1698		6533
2009	5512	7021	586	0	13,119
2010	4621	6233	1491	0	12,345
2011	5791	6157	1555	0	13,502
2012	4619	7419	3995	0	16,032
2013	1517	4390	0	0	5906
2014	3957	6414	0	0	10,370
2015	607	3391	0	0	3998
2016	973	2456	0	0	3430
*Total*	46,475	66,554	22,338	1793	137,161
German Baltic Sea (survey effort in km)					
2002	588	2609	1939	1117	6254
2003	3073	2542	0	0	5615
2004	510	2109	2874	0	5493
2005	5792	3472	2481	1111	12,856
2006	5754	1474	0	975	8203
*2007*	*No aerial surveys*				
2008	0	1572	0	816	2387
*2009*	*No aerial surveys*				
2010	1706	1728	1658	0	5092
2011	0	1709	0	0	1709
*2012*	*No aerial surveys*				
2013	0	1738	0	0	1738
*2014*	*No aerial surveys*				
2015	0	2698	0	0	2698
2016	0	1973	0	0	1973
*Total*	17,423	23,624	8952	4019	54,018

### Data preparation and analysis

Survey data were split into annual and seasonal subsets. We defined the following seasons: spring (Mar, Apr, May), summer (Jun, Jul, Aug), autumn (Sep, Oct, Nov) and winter (Dec, Jan, Feb). However, there were not enough data available for the winter season to draw conclusions on the distribution in the winter months only. We used regular observations of marine debris as well as contextual comments, such as ‘a lot of debris’. These commentaries indicated that there was a larger stretch along the transect line that was full of debris, and individual debris particles were not recorded in particular. In the absence of detailed information, we assigned these stretches post hoc a singular debris record. This certainly leads to an underestimation of available debris items but serves the purpose of at least providing minimum estimates of debris occurrence. Analyses of marine debris density were conducted on the basis of detected items per observed km (i.e. encounter rate, ER) as a measure for relative abundance of marine debris. Strip width estimation in the sense of distance sampling methodology (Buckland et al. 2001) was not possible, because declination angles needed for distance evaluation were not measured for marine debris items during our surveys. Since marine debris records were only noted as a side product rather than being in focus during these harbour porpoise monitoring surveys, angles were not measured in order to prevent distraction from harbour porpoise observations. The number of recorded general debris items, fishing-related debris items and the total survey effort (number of observed kilometres) of each subset were summarised into grid cells of 10 × 10 km (provided by the European Environmental Agency, https://www.eea.europa.eu/data-and-maps/data/eea-reference-grids-2). ERs were averaged (average encounter rate = AER) for each grid cell for fishing-related debris items (items/km), general debris items and the total number of debris items (total of the two categories). Subsequently, the ER was compared between years and seasons using standard z-test metrics. The AER was used for the spatial extent. We also calculated the ratio of fishing debris versus general debris per grid cell. In order to estimate the significance in difference, we used the ‘compare_means’ function from the ‘ggpubr’ (Kassambara 2017) R package using an unpaired *t* test metric for multiple comparisons between groups with significance level of α = 0.05.

All analyses were conducted in R Version 3.4.0 (R Core Team 2018) using the packages, sp (1.3.2) (Bivand et al. 2013, 2008; Pebesma and Bivand 2005) and maptools (Lewin-Koh and Bivand 2009).

## Results

**Fig. 3:**
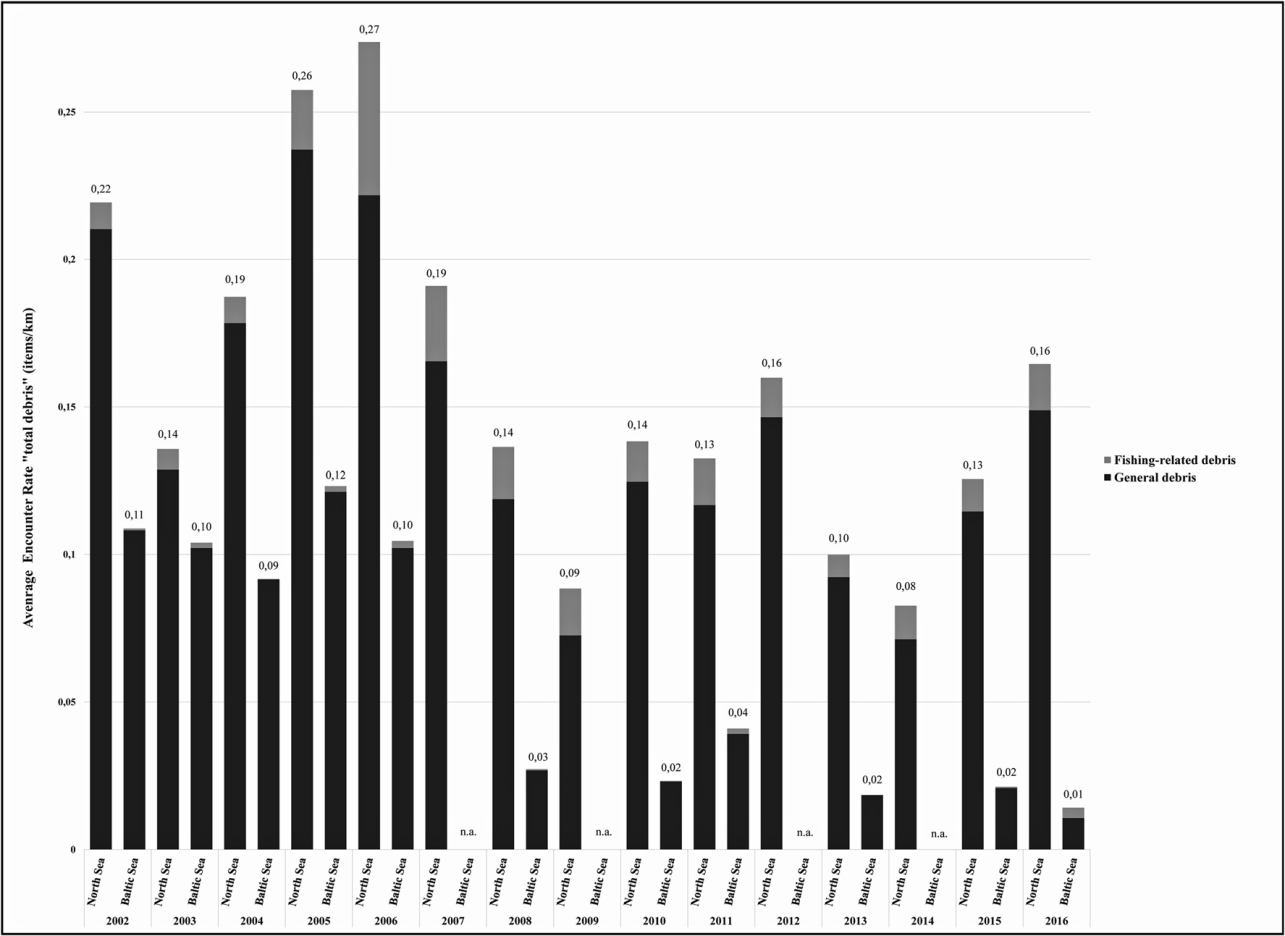
Average annual encounter rate of marine debris (total debris) in the study area of the German North Sea and Baltic Sea from 2002 to 2016 in items/km. Dashed bars indicate fishing-related debris and solid bars general debris. na = data not available, i.e. no aerial survey in these years

**Fig. 4:**
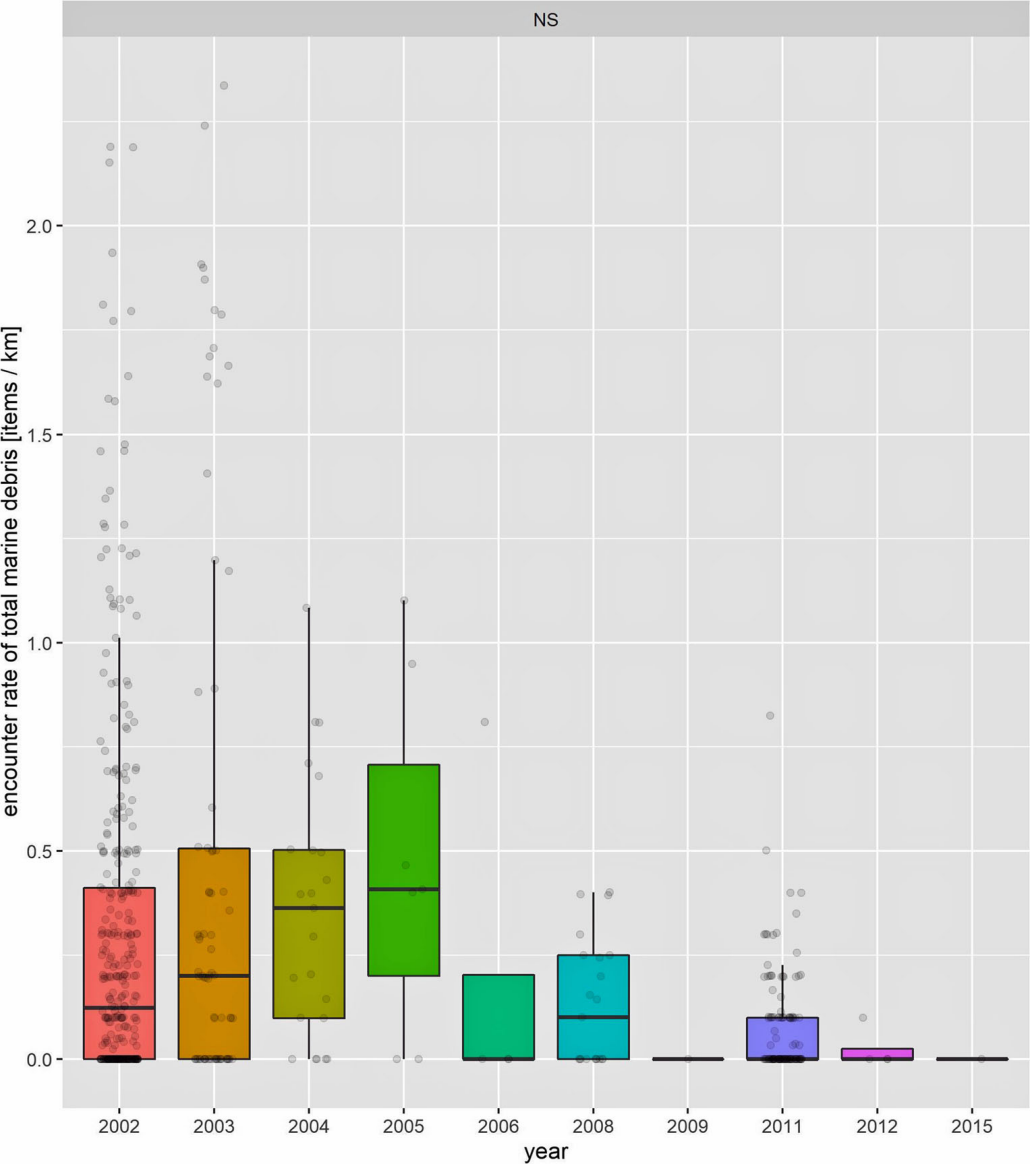
Boxplot of average encounter rate of total marine debris per year recorded during survey campaigns in the German North Sea. Individual boxplots were colourised to distinguish between years. The thick black line indicates the median of the distribution; the box itself marks the interquartile range (IQR; 25th (Q1) and 75th (Q3) percentile around the median); the vertical lines mark the maximum and minimum values (expressed as Q3 ± 1.5 × IQR); the light grey points show the actual distribution of data, scattered around the *x* axis. Note: outliers (larger/lower than maximum/minimum values) are not shown

A total of 26,512 debris items (German NS: 21,930 items, German BS: 4582 items) were recorded during the survey period. Marine debris was observed in varying quantities in all areas of the German North Sea and Baltic Sea (Figs. 1 and 2). Fishing-related debris made up 8.6% of all recorded debris items. The AER of total debris was twice as high in the North Sea (0.16 items/km) compared with the Baltic Sea (0.08 items/km). The share of fishing-related debris was higher in the North Sea (10.08%) than in the Baltic Sea (1.59%, Table 2). In the North Sea, areas with a high AER (total debris) (> 0.1 item/km) were found exclusively within the German EEZ and not within coastal areas. In the Baltic Sea, AERs of total debris were highest in the Kieler Förde and around the isle of Usedom (Fig. 1). The highest AER of total debris in the Baltic Sea was found in the eastern part in and close to the SAC ‘Odra Bank’ (EU Code/Habitats Directive: DE1652-301) (Fig. 1). Apart from the Kieler Förde and Usedom, the AERs were comparably low in coastal areas. The highest AER of total debris with more than 0.1 item/km in the North Sea was found in the north-western part of the offshore area between coastal waters (SACs Sylt Outer Reef and Borkum Reef Ground) and along the outer edge of the German EEZ (SAC Dogger Bank). Furthermore, AERs varied temporal between survey years (Figs. 3 and 4) as well as spatially within the German North Sea and Baltic Sea (North Sea, Fig. 1). The ER in the North Sea in 2005 was highest compared with all other years (Fig. 4). Data from the German Baltic Sea are not optimal for quantified visualisation, and thus, Fig. 4 concentrates on the German North Sea values.

**Table 2:** Effort, sightings and average encounter rates (AER) of marine debris in the North Sea and Baltic Sea for general and fishing-related debris items in items/km. Number of items: Number of floating marine debris items recorded on effort; Average encounter rate: average number of items per kilometre observed [items/km]; percentage: relative contribution of items to total number of items [%]; Effort: total length of transects observed on effort [km]

Sea	Category	Number of items	Average encounter rate (items/km)	Percentage (%) (no. of items)	Effort (km)
North Sea	General debris	19,719	0.14	89.92	137,161
	Fishing-related debris	2211	0.02	10.08	
	Total	21,930	0.16		
Baltic Sea	General debris	4509	0.08	98.41	54,018
	Fishing-related debris	73	< 0.01	1.59	
	Total	4582	0.08		
German waters (North Sea and Baltic Sea)	General debris	24,228	0.13	91.39	191,179
	Fishing-related debris	2284	0.01	8.61	
	Total	26,512	0.14

SACs (Fig. 1) in the German North Sea featured medium AERs (total debris) within SACs ‘Sylt Outer Reef’ (DE1209-301) and ‘Borkum Reef Ground’ (DE2104-301), ranging between 0.006 and 0.1 items/km in cells, and comparably higher AERs in SAC ‘Dogger Bank’ (DE1003-301) located further offshore, with AERs more than 0.1 item/km. In the coastal harbour porpoise protection area (Fig. 1), the AER (total debris) was lower, ranging between 0.001 and 0.03 items/km.

In the German Baltic Sea, the AERs within the SACs were generally medium, ranging between 0.001 and 0.03 items/km as well, but locally higher values up to 0.1 items/km were calculated (Fig. 1).

The contribution of fishing-related debris varied between the German North Sea and Baltic Sea (1.59% in the BS and 10.08% in the NS; Table 2). Fishing-related debris items were also ubiquitous in all surveyed areas of the North Sea, in the Baltic Sea in highest densities in the area around the SAC ‘Odra Bank’ (Fig. 2). In one specific survey year, fishing-related debris contributed up to 25% to the total amount (Baltic Sea in 2016; compare Fig. 3).

**Fig. 5:**
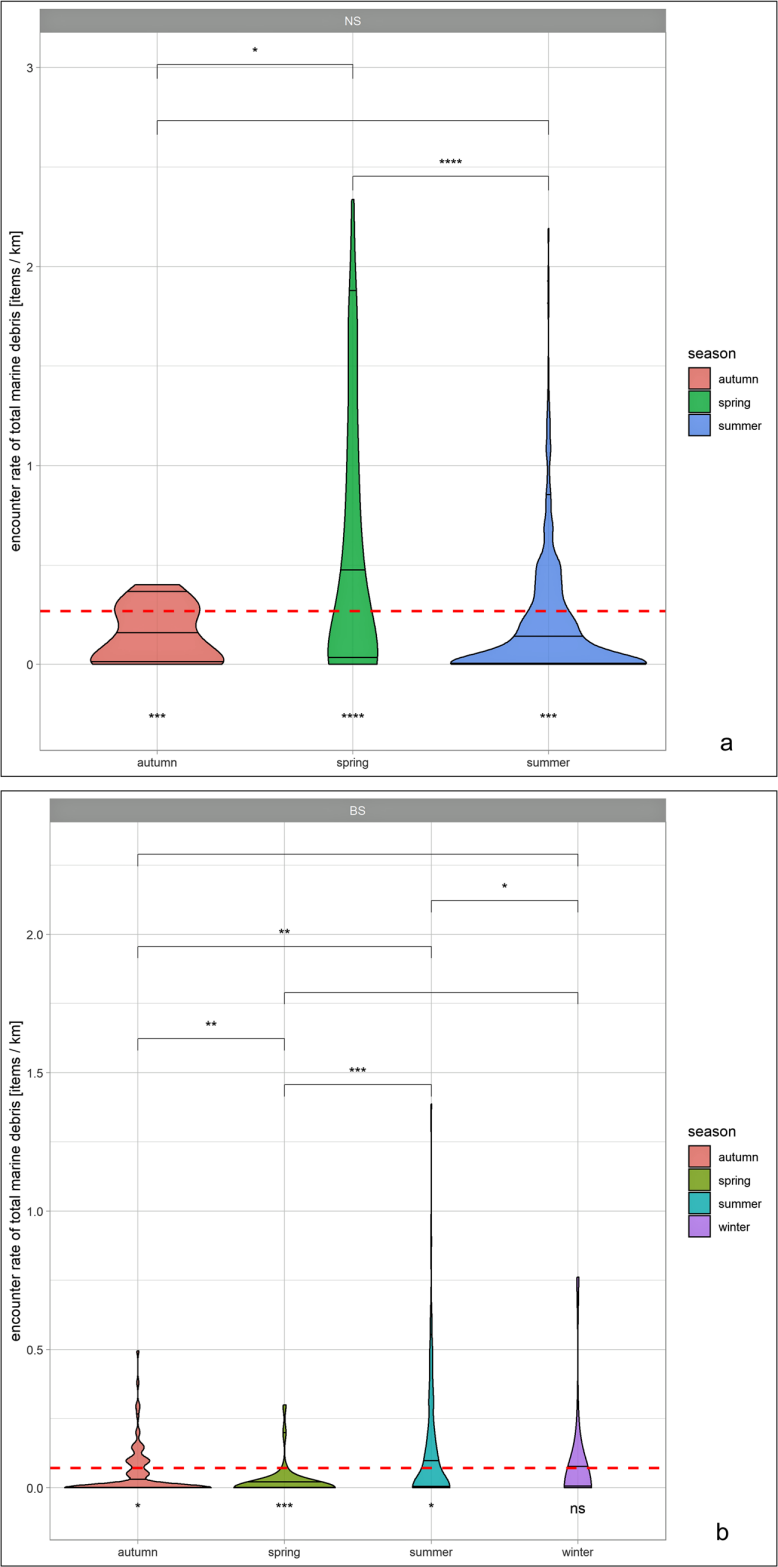
Violin plots of total marine debris encounter rates for each season for the German North Sea (**a**) and German Baltic Sea (**b**). Significance levels (α = 0.05) between seasonal means are indicated with stars and a square bracket on top of the plot, indicating the seasons that were compared. The dashed red line indicates total mean, i.e. the mean over all seasons. Correspondingly, the stars below each season indicate the significance of each seasons mean difference towards the overall mean, with ns not significant

The ER (total debris) in the North Sea ranged between 0 and 0.1 items/km during spring, summer and autumn. The ER in spring ranged between 0 and 0.02, in summer between 0 and 0.8 and in autumn between 0 and 1.0. Most values are below 0.1 items/km (Fig. 5).

In both the German North Sea and Baltic Sea, the AERs (total debris) of marine debris peaked in spring. Especially in the Baltic Sea, the AER (total debris) in spring was up to three times larger than in the other seasons (Table 3). The ER is significantly higher in spring compared with the summer season both in the North Sea (*p* < 0.0001) and Baltic Sea (*p* < 0.01) (Fig. 5).

**Table 3:** Seasonal average encounter rate (AER) of marine debris in the German North Sea and Baltic Sea for general and fishing-related debris items in items/km. Number of items: Number of floating marine debris items recorded per survey effort; Average encounter rate: average number of items per kilometre observed [items/km]; percentage: relative contribution of items to total number of items [%]; Effort: total length of transects observed on effort [km]

Sea	Season	Category	Number of items	Average encounterrate (items/km)	Percentage(%; items to totalnumber of items)	Effort (km)
North Sea	*Spring*	General debris	7775	0.1673	89.77	46,463
		Fishing-related debris	886	0.0191	10.23	
		Total	8661	0.1864		
	Summer	General debris	9129	0.1372	89.25	66,554
		Fishing-related debris	1100	0.0165	10.75	
		Total	10,229	0.1537		
	Autumn	General debris	2546	0.114	92.18	22,337
		Fishing-related debris	216	0.0097	7.82	
		Total	2762	0.1236		
	Winter	General debris	269	0.15	96.76	1793
		Fishing-related debris	9	0.005	3.24	
		Total	278	0.155		
Baltic Sea	*Spring*	General debris	2447	0.1404	98.04	17,423
		Fishing-related debris	49	0.0028	1.96	
		Total	2496	0.1433		
	Summer	General debris	1318	0.0558	98.43	23,623
		Fishing-related debris	21	0.0009	1.57	
		Total	1339	0.0567		
	Autumn	General debris	554	0.0619	99.82	8952
		Fishing-related debris	1	0.0001	0.18	
		Total	555	0.062		
	Winter	General debris	190	0.0473	98.96	4018
		Fishing-related debris	2	0.0005	1.04	
		Total	192	0.0478		

Month with highest AER in italics

## Discussion

In this study, data on marine debris collected during aerial surveys for harbour porpoises were successfully analysed for distributional patterns of marine debris, providing the first comprehensive overview for German waters. Our analyses detected areas with high marine debris occurrence areas within the North Sea and Baltic Sea, which allowed for temporal as well as spatial assessments of debris distributions over the entire German North Sea and German Baltic Sea and enabled an estimation of debris loads in SACs. Our results demonstrate the gain of information on marine debris distribution that can be obtained from data collected opportunistically during surveys with another target.

Comparing the results presented here with results of earlier local and smaller-scale assessments in German waters on marine debris distribution supports the validity of the distribution patterns identified based on aerial survey data. Dixon and Dixon (1983) also reported the highest amounts of debris in areas far from the coast (within the German Exclusive Economic Zone, EEZ) based on shipboard survey data. Shipboard surveys from 2006 to 2008 (Thiel et al. 2011) as well as follow-up data until 2016 (Gutow et al. 2018) indicated a high density of plastic objects off the East Frisian Islands. Furthermore, the analysis for 2016 revealed high densities in offshore waters, between the SACs Sylt Outer Reef, Borkum Reef Ground and the Dogger Bank, which correspond with our results. In comparison with these previous surveys, our data covered a much wider spatial area, providing a large-scale overview. Therefore, it is reassuring that local densities seem to be in line with estimates from ship-based surveys, validating our approach and estimates for areas which have not been surveyed before.

In the North Sea, inputs of the Rhine River largely end up in offshore waters of the German EEZ (Gutow et al. 2018) and have already been suspected to be a major contributor to marine micro plastic pollution in the North Sea (Lebreton et al. 2017; Mani et al. 2015; OSPAR 2000). The introduction of a similarly significant amount of larger items is likely and may explain the higher AER (total debris) in the southern North Sea which was also shown in earlier evaluations (Dixon and Dixon 1983). In the Baltic Sea, objects entering from land-based sources will rather soon be washed up on beaches close by (Schernewski et al. 2017). Therefore, a contribution of land-based sources to floating marine debris accumulated offshore is less rather likely for the Baltic Sea.

Our ERs are lower than those from other European marine areas. In Portuguese waters, ERs of up to 11.51 items/km were observed during shipboard surveys (Sá et al. 2016) and up to 1.54 items/km in the Ligurian Sea in 1997 (Mediterranean) (Aliani et al. 2003). This may point to a lower debris burden in German waters. The North Sea and Baltic Sea are categorised as two of the most anthropogenically impacted seas in the world (Halpern et al. 2008, 2015). As areas of special risk, due to their semi-enclosed locations, dumping debris at sea in the North Sea and Baltic Sea has been regulated since 1973 by the International Convention for the Prevention of Pollution from Ships (MARPOL, Annex V). As ‘particularly sensitive sea areas’ (PSSA), the input of plastic garbage and fishing gear into the Wadden Sea located in the North Sea and the entire Baltic Sea is strictly prohibited (IMO 2018). However, low encounter rates may also be due to generally lower detection rates during aerial surveys compared with shipboard surveys, due to the high survey speed and observing altitude. Especially the proximity to the surface during ship surveys results in the fact that smaller items are detectable compared with aerial surveys (see Sá et al. (2016)).

A quantitative comparison with results from other studies is difficult, because most studies conducted were ship-based. Furthermore, most studies report marine debris as densities in items/area (km^2^). Our surveys did not allow for estimation of the effectively observed area for marine debris. While distance sampling surveys always estimate the effectively covered strip width (esw), during our surveys, this was only done for the target species harbour porpoise. Strip width estimation is target-specific and requires measurements of the distance to the transect line for all detected objects/targets. Since debris items were recorded as an add-on during a marine mammal survey, no declination angle was recorded for debris items, as this would have drawn attention away from harbour porpoise observation. Debris information from our surveys can thus only be reported as number of items per observed kilometre, representing a relative measure rather than true densities.

Another restriction of opportunistic data collection during aerial surveys lies in a limited chance of identifying objects in detail. While mostly it is possible to identify objects as ‘non-natural’ and thus as debris, specifying objects from air may be difficult at times. Therefore, in comparison with ship-based surveys or beach collection, aerial surveys provide less opportunity to investigate debris composition. Nevertheless, a basic distinction between general debris and fisheries-related debris is possible, as demonstrated in our study. In fact, we detected a considerable share of marine debris to be fisheries related. Fishing gear is regularly lost during fishing operations. Furthermore, illegal discard of old fishing gear contributes to the category of ‘abandoned, lost or otherwise discarded fishing gear (ALDFG)’ (Macfadyen et al. 2009). Worldwide, ALDFG is a significant component of marine debris posing an especially high risk for marine fauna, because of their inherent entangling characteristics. In our study, fishing-related debris made up 10.8 and 1.59% of all recorded debris items in the North Sea and Baltic Sea, respectively. In some years, up to 25% (Baltic Sea in 2016) of all recorded debris was categorised as fisheries-related debris. Possibly, not all fisheries-related items will have been identified as such; therefore, the estimated share of fisheries related debris most likely represents an underestimate. These data demonstrate the prevalence of particularly harmful marine debris items floating in German waters, which should give reason for concern.

Another reason for concern arises from medium to high concentrations of marine debris, including fisheries-related debris, identified in SACs. Marine debris poses a threat to species for which the areas have been established. In the case of marine mammals in German waters, harbour porpoises and seals are at risk of entanglement in and ingestion of marine debris (Unger et al. 2017; Unger et al. 2016), a threat that is unacceptable in areas designed for the protection of these animals.

Despite some limitations, these data are valuable for a range of possible analyses, with the added benefit of having been collected at no further cost and very limited additional effort. The power of the aerial dataset lies in the large spatial and temporal coverage providing a large-scale overview not available from any other source. Our study is the first to present an overview of marine debris distribution for the entire German North Sea and Baltic Sea. The analysis conducted showed that aerial surveys are suitable for observing distribution patterns. Furthermore, the dataset provides a good basis for further analyses of sources and entry ports of marine debris into the seas, as well as for analyses of transport mechanisms within the seas. Our data could be used in wind and current models predicting particle dispersal as shown in Neumann et al. (2014). Furthermore, our results can be analysed in combination with debris collections on beaches in order to combine information on debris composition and distribution, as well as transport at sea. Despite some minor drawbacks, the collected data provide invaluable information on pollution loads, required to meet assessment criteria for management frameworks such as the MSFD. Based on our experience, we recommend that data collection on marine debris be mandatory during monitoring surveys for marine top predators throughout Europe, in order to obtain a possibility to assess marine debris distribution on a large scale.
